# Invasive Agricultural Pest *Drosophila suzukii* (Diptera, Drosophilidae) Appeared in the Russian Caucasus

**DOI:** 10.3390/insects11110826

**Published:** 2020-11-23

**Authors:** Andrzej O. Bieńkowski, Marina J. Orlova-Bienkowskaja

**Affiliations:** A.N. Severtsov Institute of Ecology and Evolution, Russian Academy of Sciences, 119071 Moscow, Russia; bienkowski@yandex.ru

**Keywords:** invasive species, spotted-wing drosophila, quarantine pest, European Russia

## Abstract

**Simple Summary:**

Spotted-wing drosophila, *Drosophila suzukii*, is one of the most important invasive pests of fruit and wine production worldwide. This species feeds on cherry, raspberry, blackberry, blueberry, strawberry, peach, plums, grape, and other soft fruits. It causes significant damage because, unlike most other *Drosophila* species, it oviposits and feeds on healthy fruits. *Drosophila suzukii* is native to East Asia and has been rapidly spreading through Europe, where it is considered a quarantine pest, since 2008. Herein, we report the first records of spotted wing drosophila in European Russia. In 2017 and 2020, we placed baited traps in different districts of the resort city of Sochi (Black sea Coast of the Caucasus, Krasnodar Territory, Russia) and collected 49 adults of this species. They were identified by the typical female ovipositor, spotted wings of the males, and other characters. Krasnodar Territory is one of the main fruit-producing regions of Russia. Therefore, populations of this pest should be monitored and measures to minimize economic damage should be taken.

**Abstract:**

Spotted-wing drosophila *Drosophila suzukii* (Matsumura) is one of the most important invasive pests of fruit and wine production worldwide. This species feeds on *Prunus* spp., *Rubus* spp., *Fragaria* spp. (Rosaceae), *Vaccinium* spp. (Ericaceae), *Vitis* spp. (Vitaceae), and other soft fruits. It causes significant damage because, unlike most other *Drosophila* species, it oviposits and feeds on healthy fruits. *Drosophila suzukii* is a quarantine pest that is included on the European and Mediterranean Plant Protection Organization (EPPO) A2 List. This species is native to East Asia and has been rapidly spreading through Europe since 2008. Herein, we report the first records of *D. suzukii* in European Russia. In 2017 and 2020, we placed baited traps in different districts of the resort city of Sochi (Black sea Coast of the Caucasus, Krasnodar Territory, Russia). Three specimens of *D. suzukii* were collected in June 2017, two specimens in September 2017, and 44 specimens in September 2020. Specimens were identified by the typical female ovipositor, spotted wings of the males, and other morphological characters. Krasnodar Territory is one of the main fruit-producing regions of Russia. Therefore, populations of this pest should be monitored and measures to minimize economic damage should be taken.

## 1. Introduction

The rapid spread of invasive pests is a great economic and ecological problem of the 21st century [1]. An unusually high number of invasive pests new to European Russia were first detected in the subtropics of the Russian Caucasus, namely in the city of Sochi in the last 10–20 years. This region is associated with an increase in international trade, especially bulk imports of planting material from W. Europe (mainly Italy), and imports of various products from East Asia, including China. The city of Sochi with its seaport, railway station, and airport is a major center of international trade, and the favorable subtropical climate contributes to the establishment of alien insects [2].

Spotted-wing drosophila, *Drosophila suzukii* (Matsumura, 1931), is one of the most important invasive pests of fruit and wine production worldwide [3]. This species damages cherry, peach, and plums (*Prunus* spp., Rosaceae); raspberry and blackberry (*Rubus* spp., Rosaceae), strawberry (*Fragaria* spp., Rosaceae); blueberry (*Vaccinium* spp., Ericaceae); grape (*Vitis* spp., Vitaceae); and other soft fruits. *Drosophila suzukii* has a wide host range compared to many other drosophila, and in Western Europe (Italy, Switzerland, the Netherlands), *D. suzukii* has been recorded to feed on 84 species of plants in 19 families [4]. The economic damage caused by this species has prompted numerous studies regarding its biology and control; for instance, a special issue of the *Journal of Pest Science* was devoted to this species [5]. The harmful effects of fruit flies are not limited to just economic damage, and *Drosophila* larvae have been reported to cause intestinal myiasis in humans [6]. Unlike other fruit flies, *D. suzukii* will oviposit and develop in healthy ripening soft fruits. This increases the danger of *D. suzukii* to human health, as the consumption of what appear to be normal fruits that contain drosophila larvae can lead to conditions such as intestinal myiasis.

*Drosophila suzukii* is native to East Asia and has spread to Europe, Asia, Africa, the Americas, and Oceania [7]. It was first recorded in Europe, particularly in Spain (Barcelona), in 2007 [8]. It then quickly spread to much of Europe. In 2009, it was recorded in France [8]; in 2011 in Austria [9], Belgium [10], Croatia [11], Italy [12], Slovenia [13], and Switzerland [14]; in 2012 in Germany [15], Great Britain [16], Hungary [17], and the Netherlands [18]; in 2013 in Montenegro [19] and Romania [20]; in 2014 in Bosnia and Herzegovina [21], Greece [22], Poland [23], Serbia [24], and the Czech Republic [25]; and in 2016 in Bulgaria [26] and Cyprus [27]. In the continent of North America, *D. suzukii* was first found in 2008 in California (USA), and it has spread to other parts of the USA, Mexico, and Canada [28]. In South America, it was recorded in 2013 in southern Brazil, and it later spread to much of the rest of South America [29]. This pest continues to spread in Eurasia; in 2014, it was first found in Turkey [30], and in 2015, it was first reported from Iran [31].

Ecological modeling of the potential range of *D. suzukii* has shown the suitability of temperate and subtropical latitudes of the Northern and Southern Hemispheres in Europe, North and South America, and Asia. There is the potential that it will also spread south into much of Africa and invade Australia [28]. It has been determined that limited precipitation and low temperatures are the main factors limiting the spread of *D. suzukii*. This species requires sufficient humidity and mild winters to establish in new regions [32]. *Drosophila suzukii* has been included in the European and Mediterranean Plant Protection Organization (EPPO) A2 List of quarantine pests since 2011 [33].

*Drosophila suzukii* is also spreading in the Black Sea region. In 2014, it was first recorded for Crimea [34] and East Turkey [30] and three years later, in 2017, in the Adjara Region in southeastern Georgia [35]. Based on these data, we hypothesized that *D. suzukii* could also spread to the Black Sea Coast of the Russian Caucasus. We performed field surveys that confirmed this hypothesis. Herein, we report the first records of *D. suzukii* in European Russia, namely in Sochi. This city is located a great distance from the previously known localities of *D. suzukii* in the surrounding regions: About 500 km from Crimea, about 450 km from east Turkey, and about 250 km from Adjara.

## 2. Materials and Methods

The survey was conducted from 4 to 19 June 2017 and 18 to 30 September 2017 in the Central District of Sochi, and from 16 to 26 September 2020 in the Adler District of the city.

The collecting site, the resort city of Sochi, is in the Krasnodar Territory in the south of European Russia on the Black Sea Coast of the Russian Caucasus. We placed the traps in the Central and Adler Districts of Sochi. Four types of traps were used: (1) Traps made of plastic bottles baited with a mixture of commercially available red wine and vinegar, which were recommended by Cini et al. [3] for detection of *D. suzukii*; (2) traps (plastic bottles) baited with ripe local grapes; (3) pitfall traps baited with vinegar; (4) barrier traps, commonly used to collect flying bark beetles, baited with a mixture of commercially available red wine and vinegar. Traps were placed in various types of plant associations: Ornamental plantings, areas of natural forest plantations, and ruderal vegetation (especially near fruit markets, food dumps) in settlements, mixed forest, and forest edges in the foothills.

Photographs of the habitus and structural details were made by a Nikon D 90 digital camera, combined with Tamron SP 70–300 mm F/4-5.6 and inverted Nikon AF NIKKOR 28–105 mm 1:3.5–4.5 D lenses.

## 3. Results

Adult specimens of *D. suzukii* were collected in three localities: Sochi, Zavokzalny Distr., near the Sochi railway station, edge of deciduous forest, 43.589691 N, 39.728756 E, trap type 1, 4–19 June 2017, 1 male; Sochi, Svetlana Distr., Serafimovicha Street, 43.578291 N, 39.735929 E, trap type 2, 18–30 September 2017, 1 mature female, 1 female from puparium; Sochi, Adler Distr., Moldovka Vill., hill, 100 m ASL, edge of deciduous forest, trap type 3, 6 males, 12 females, trap type 4, 8 males, 18 females, 43.457627 N, 39.947431 E, 16–26 September 2020. The material is kept in the collection of the first author, and 10 specimens will be transferred to the All-Russian Center for Plant Quarantine (VNIIKR).

**Figure 1:**
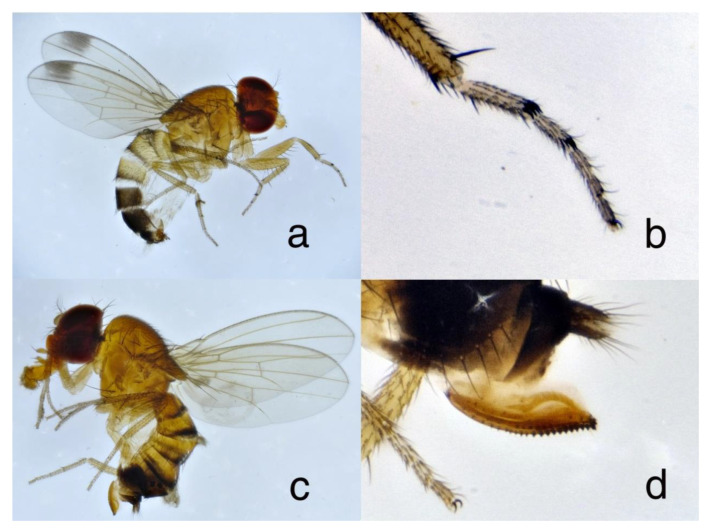
*Drosophila suzukii*, European Russia, Sochi city. (**a**) Male, total view; (**b**) male, fore tarsus; (**c**) female, total view; (**d**) female, ovipositor.

Several males and females were dissected and mounted in Berlese medium for microscopic examination and photography, while others were preserved in alcohol. Specimens were identified by referring to Hauser [36] and Calabria et al. [8]. Both sexes: Body 2–3 mm long (female, some larger than male), pale brown with eyes red, with abdomen partly darkened dorsally. Male: One black spot at the apical part of each wing and two short black combs at the apex of 1st and 2nd fore tarsal segments (one comb on each segment). Female: Large ovipositor with many dark sclerotized teeth, ovipositor much longer (at least 6×) than spermatheca diameter (Figure 1). The combination of black wing spot and black tarsal combs in male does not occur in any other Holarctic drosophila.

## 4. Discussion

The specimens were captured in different districts of the city and in different years and months, indicating that the species has established in the region. It is probable that the spotted-wing drosophila could have been unintentionally introduced to the Caucasus with imported fruits. Fruit production and wine production are important aspects of the economy in the Russian Caucasus region, so the establishment of *D. suzukii* could cause serious economic losses. Quarantine measures should be taken to prevent dispersal of this pest in the surrounding regions and especially in the Caucasus.

It is not surprising that the new invasive pest *D. suzuki* was found at the Black Sea Coast of the Caucasus. This region is a hotspot for biological invasions of insects in Russia. In particular, about 30 arboreal pests were discovered there in just the past 10 years (2010–2019): *Paysandisia archon* Burmeister (Lepidoptera: Castniidae), *Rhynchophorus ferrugineus* (Olivier) (Coleoptera: Rhynchophoridae), *Ceroplastes ceriferus* (Fabricius) (Homoptera: Pseudococcidae), *Glyphodes pyloalis* Walker (Lepidoptera: Crambidae), *Dasineura gleditchiae* (Osten Sacken) (Diptera: Cecidomiidae), *Lamprodila (Palmar) festiva* (Linnaeus) (Coleoptera: Buprestidae), *Phyllonorycter robiniella* (Clemens) (Lepidoptera: Gracillariidae), *Halyomorpha halys* (Stål) (Heteroptera: Pentatomidae) and others [2,37]. The high number of invasions to the Black Sea Coast of the Caucasus is probably connected with the unique combination of factors that facilitate biological invasions. First, the climate in this region is wet subtropical, and therefore favorable to the establishment of alien insect species from other parts of the world with a similar climate. Second, the Black Sea Coast of Russia is one of the main sea resort regions of Russia. Trains and airplanes from all over the country bring there about ten million people every year. As such, the probability of the unintentional introduction of pests from different areas to the resort region is very high. Insect pests could be introduced as stowaways in transport, as contaminants with food products and other goods of animal and plant origin, and especially with planting material. It should be noted that the urban plantings in the city of Sochi and other resort cities of the region consist mainly of exotic plant species. Large numbers of seedlings, bulbs, and seeds are imported to the region every year. In particular, intensive landscaping of the streets and parks was conducted in preparation for the Olympic Games in 2014 [2]. 

It is interesting that *D. suzuki* has become a global invader very quickly. In particular, in less than 10 years after the first record in Europe, this species has spread over much of Europe, from the Netherlands in the north to the Balkan Peninsula in the south. It has also spread to West Asia, North America and South America, Africa, and Oceania [7]. Our findings in the Russian Caucasus demonstrate that this species could be present in many other regions and stay unnoticed there for a long time. It indicates that the real range of the species could be more extensive than it is believed now, and special surveys should be made in different regions of the world to discover new populations. It is not difficult to find the spotted-wing drosophila if it is established in the region. A variety of simple types of traps bated with wine, vinegar, or grape are shown to be effective to collect drosophila.

The rapid spread of invasive pests is usual in the 21st century. It is one of the most negative consequences of globalization. Development of efficient transportation methods and the intensification of international trade have largely eliminated the geographical barriers for insect spread. The existing system of plant quarantine was unable to prevent the spread of *D. suzuki* and many other invasive pests. It should be considered that as biological invasions are global processes, regional plant quarantine services cannot entirely prevent the spread of invasive pests. Therefore, we believe that a global system of insect fauna monitoring should be developed in order to ensure ecological and economic security from invasive insect pests. 

## 5. Conclusions

1. *Drosophila suzukii* (Diptera, Drosophilidae), an invasive pest of fruit and wine production, has established in the Russian Caucasus.
2. Our finding suggests that the Black Sea Coast of the Caucasus is a hotspot of biological invasions for insect pests to European Russia. There needs to be more attention given to invasive species in this region.
3. Spotted-wing drosophila shows that if an insect species has established outside its native range, it could spread over different continents and become a global invader very quickly (in just ten years).
4. The spotted-wing drosophila stayed unnoticed for several years after its establishment in the Caucasus. Therefore, it is quite possible that it has already established and remained undetected in other regions, and the current range of this invasive pest is more extensive than documented. To reveal the real range of this pest and to mitigate potential economic losses, surveys for this species should be conducted where possible.
5. The existing system of plant quarantine could not prevent the spread of *D. suzukii* neither within Europe nor on a global scale, as is the case with many other invasive pests in the last 10–20 years. It seems that a new global system of insect pest monitoring should be developed.
